# Evaluation of the Cancer Transition Theory in the US, Select European Nations, and Japan by Investigating Mortality of Infectious- and Noninfectious-Related Cancers, 1950-2018

**DOI:** 10.1001/jamanetworkopen.2021.5322

**Published:** 2021-04-12

**Authors:** Omer Gersten, Magali Barbieri

**Affiliations:** 1 Nu-Trek, San Diego, California; 2 Department of Bioinformatics and Biostatistics, University of California, San Diego Extension, La Jolla; 3 Department of Demography, University of California, Berkeley; 4 French Institute for Demographic Studies, Paris, France

## Abstract

**Importance:**

Despite cancer being a leading cause of death worldwide, scant research has been carried out on the validity of the cancer transition theory, the idea that as nations develop, they move from a situation where infectious-related cancers are prominent to one where noninfectious-related cancers dominate.

**Objective:**

To examine whether cancer transitions exist in the US, select European countries, and Japan.

**Design, Setting, and Participants:**

In this cross-sectional study, annual cause-of-death data from the 1950s to 2018 for the US, England and Wales, France, Sweden, Norway, and Japan were extracted from the Human Mortality Database and the World Health Organization (WHO). Statistical analysis was performed from April 2020 to February 2021.

**Main Outcomes and Measures:**

Age-standardized death rates for all ages and both sexes combined were estimated for cancers of the stomach, cervix, liver, lung, pancreas, esophagus, colorectum, breast, and prostate.

**Results:**

The results of the analysis show that for all countries in this study except for Japan, mortality from infectious-related cancers has declined steadily throughout the period, so that by the end of the period, for Norway, England and Wales, Sweden, and the US, rates were approximately 20 deaths per 100 000 population. Regarding noninfectious-related cancers, at the beginning of the period, all countries exhibited an increasing trend in rates, with England and Wales having the greatest peak of 215.1 deaths per 100 000 population (95% CI 213.7-216.6 deaths per 100 000 population) in 1985 followed by a decline, with most of the other countries reaching a peak around 1990 and declining thereafter. Furthermore, there is a visible crossover in the trends for infectious-related and noninfectious-related cancers in Japan and Norway. This crossover occurred in 1988 in Japan, when the rates for both types of cancers stood at 116 per 100 000 population (95% CI, 115.0-116.5 per 100 000 population), and in 1955 in Norway, when they passed each other at 100 per 100 000 population (95% CI, 96.4-105.3 per 100 000 population).

**Conclusions and Relevance:**

In this cross-sectional study, the findings suggest that cancer mortality patterns parallel the epidemiological transition, which states that as nations develop, they move from a stage where infectious diseases are prominent to one where noninfectious diseases dominate. An implication is that the epidemiological transition theory as originally formulated continues to be relevant, despite some researchers arguing that there should be additional stages beyond the original 3.

## Key Points

**Question:**

As nations develop, do they experience a systematic pattern in cancer trends by type, distinguishing between infectious-related and noninfectious-related cancers?

**Findings:**

This cross-sectional study of 6 countries’ cancer mortality data from 1950 to 2018 found that a crossover in trends between the 2 main types of cancers (infectious-related and noninfectious-related) took place around 1990 in Japan and in the mid-1950s in Norway. For the other countries in the study, the trends in the 2 types of cancers do not intersect as they do for Japan and Norway, but those other nations still exhibit a cancer transition with declining rates of infectious-related cancers and rates of noninfectious-related cancers initially increasing, before eventually declining.

**Meaning:**

These findings support the theory that cancer transitions are occurring in the US, select European nations, and Japan.

## Teaser

This cross-sectional study examines the incidence of infectious- and noninfectious-related cancers in the US, select European countries, and Japan from 1950 to 2018.

## Introduction

Cancer is one of the leading causes of death worldwide, and because of marked declines in cardiovascular disease in several countries, cancer has become the leading cause of death in many high-income populations.^1,2,3^ Although the important role of infection in causing certain cancer types is established and quantified (2.2 million cancer cases were attributable to infection in 2018),^4,5,6^ the view of cancer as a prime example of a “degenerative and man-made disease”^7^ remains.^8,9^ Indeed, most global reports, including those of the World Health Organization (WHO), categorize cancer as a key noncommunicable disease.^10^

The article by Omran^7^ on the epidemiological transition has influenced a number of academic fields.^11,12^ In his article, Omran^7^ postulated that in the third and last stage of the transition, noncommunicable and chronic ailments such as heart disease and cancer replace acute, infectious diseases as prime killers. In this age of degenerative and lifestyle-related diseases, infectious diseases experience a progressive decline but do not disappear entirely.^7^

Omran’s theory,^7^ however, is overly simplified because it overlooks the fact that infection is often an important cause of cancer.^13^ For instance, we now know that the bacterium *Helicobacter pylori* (*H pylori*), hepatitis B and C viruses, and human papillomavirus are important causes of cancers of the stomach, liver, and cervix, respectively. The number of new cancer cases in 2012 attributable to infectious agents was about 79% for stomach cancer, 73% for liver cancer, and 100% for cervical cancer.^13^

Gersten and Wilmoth^14^ first introduced and developed the concept of the cancer transition, which they meant to be analogous to Omran’s epidemiological transition. The authors analyzed cancer trends in Japan from 1951 until 1997 and found that cancers associated with infectious causes were declining, whereas those associated with noninfectious causes were increasing. There has thus been a playing out of the epidemiological transition within the broad and complex set of diseases that define cancer.

Work investigating and attempting to extend the cancer transition theory has been limited, perhaps numbering no more than a few articles. However, Knaul and colleagues^15^ have seriously engaged with the theory and presented cross-sectional data from GLOBOCAN (2008)^16^ suggesting that cancers associated with infectious causes become less prominent as many nations move along the gradient from less to more developed. A similar finding has been reported by Fidler and colleagues^17^ in a more recent article using a different data set.

Bray et al^4^ also extend the cancer transition literature. They analyzed 4 levels of the Human Development Index—a composite indicator of life expectancy, education, and gross domestic product per person—to highlight cancer-specific patterns in 2008 and trends from 1998 to 2002, and to produce future burden scenarios for 2030. According to Bray et al, “The reduction in infection-related cancers seems to be offset by concomitant increases in cancers related to a Westernization of lifestyle and changes in tobacco consumption and the effect on lung and other cancers.”^4^

The aim of the present study is to contribute to the nascent literature on the cancer transition theory, and in so doing, examine the relevance of the epidemiological transition theory to understand current mortality trends. This article focuses on evidence of cancer transition in the US, select European nations, and Japan, differentiating between cancers associated with an infectious cause and other cancers.

## Methods

Because all of the statistical information used in this cross-sectional study is derived from publicly available, deidentified, aggregated death records, the University of California, Berkeley institutional review board has determined that this study is exempt from review and does not require informed patient consent. This study followed the Strengthening the Reporting of Observational Studies in Epidemiology (STROBE) reporting guideline.

We selected the countries in our analysis according to the availability, detail, and quality of their cause-of-death records and how far back the data series extend. Annual cause-of-death data were obtained directly from the WHO Mortality Database^18^ and combined with life table series from the Human Mortality Database^19^ to compute age-standardized death rates for common types of cancer in 6 countries during extended periods: the US (1959-2017), England and Wales (1950-2018), France (1958-2016), Norway (1951-2016), Sweden (1952-2016), and Japan (1950-2016).

The mortality and population data from the WHO Mortality Database and the Human Mortality Database used for the numerators and denominators of the rates are derived from the information available on the death certificates and in the census for the entire population of each country. The European 2013 standard population was used to account for differences in age structure across the 6 countries.

Our analysis presents results for all ages and both sexes combined and for 9 cancers grouped according to their known causes, separating those associated with infectious causes from those associated with noninfectious causes. Cancers associated with infectious causes include stomach, liver, and cervical cancer, which together accounted for 19.7% of worldwide cancer deaths in 2018.^1^ Concerning the cancers associated with noninfectious causes, those cancers (and their main causes) are lung (smoking),^20^ pancreatic (smoking),^21,22^ esophageal (smoking and drinking),^23,24^ breast (reproductive or hormonal and physical activity),^25,26^ colorectal (Western lifestyle),^27,28^ and prostate (age, ethnicity, and family history).^29,30,31^ Together, these noninfectious-related cancers accounted for 47.6% of worldwide cancer deaths in 2018.^1^

The *International Classification of Diseases (ICD)*, implemented by all study countries during the period under consideration, has gone through numerous revisions, from the sixth in 1950 to the tenth currently. These *ICD* revisions introduce some disruptions in the data series as illustrated by trends in the death rates from liver cancers in both Japan and France. However, as documented in the literature, cancer as a disease is much better defined than many others and these disruptions are small enough that a general picture of the overall trends emerges very clearly.^32,33^ Similarly, differences in diagnostic and coding practices do not appear to be large enough across countries to bias international comparisons in cancer mortality trends.^34,35^ We define the cancer transition as the process through which a country’s population moves from a situation where rates of infectious-related cancers are greater than those of noninfectious-related cancers, to the converse situation where rates of noninfectious-related cancers are higher.

### Statistical Analysis

The 95% CIs for the age-standardized death rates were calculated using the Keyfitz formula.^36^ Statistical analysis was performed using RStudio statistical software version 1.3.959 (Integrated Development for R, PBC) from April 2020 to February 2021.

## Results

**Figure 1.**
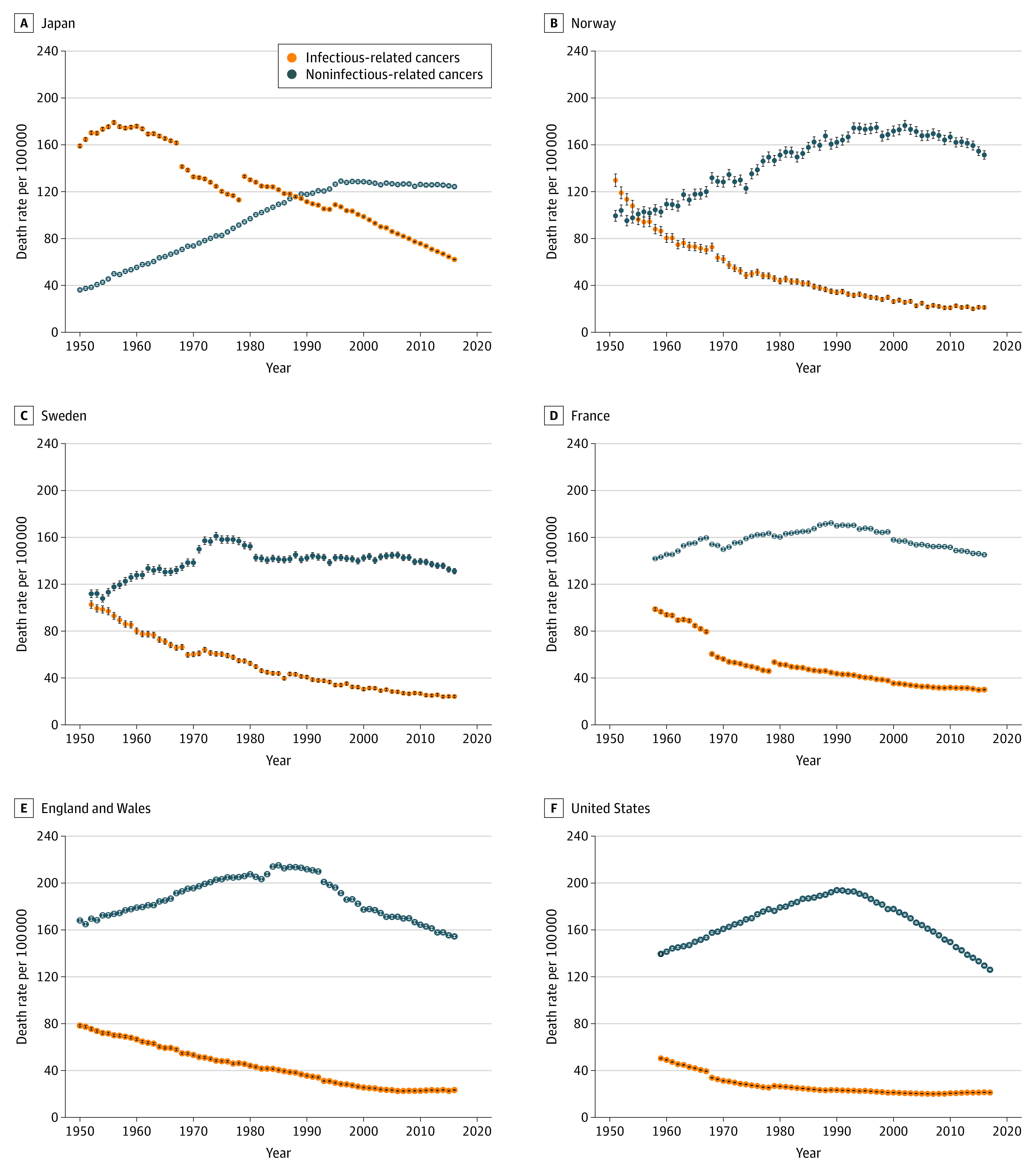
Age-Standardized Death Rates for Infectious- vs Noninfectious-Related Cancers for Both Sexes Combined in Japan, Select European Nations, and the US Error bars denote 95% CIs.

**Figure 2.**
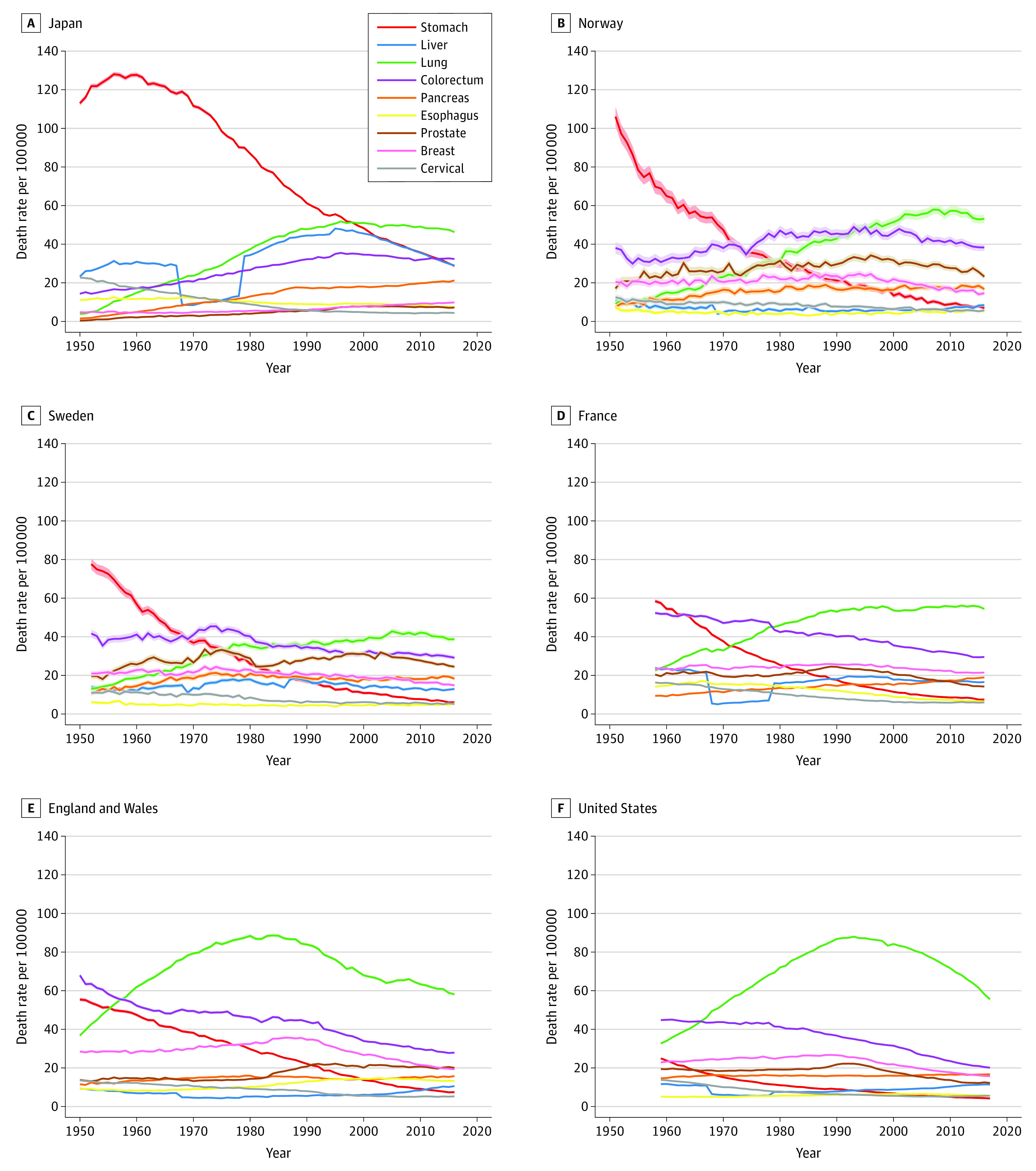
Age-Standardized Death Rates for the Most Common Cancers Over Time for Both Sexes Combined, in Japan, Select European Nations, and the US Shaded regions denote 95% CIs.

Trends in the age-standardized death rates from infectious-related cancers and noninfectious-related cancers in the 6 study countries are presented on Figure 1. Trends in the specific cancers making up each of the 2 categories (infectious-related and noninfectious-related cancers) are presented in Figure 2. The 95% CIs for the rates are indicated in the figures. Note that because the rates are calculated for each national population, size effects are small and the 95% CIs are hardly visible except for the 2 countries with the smallest populations, Sweden and Norway. An Excel file with 1 worksheet per country provided as the Supplement contains the exact rates for infectious-related cancers, noninfectious-related cancers, and all cancers in the study and their 95% CIs, as well as the percentage share of each specific cancer within its category (infectious-related and noninfectious-related cancers). A comparison between the epidemiological and cancer transition theories is presented schematically in the Table.

**Table.** Comparison of the Epidemiological Transition and Cancer Transition Theories

	Before: infectious disease	After: noninfectious disease
Epidemiological transition	Malaria	Cancer
	Tuberculosis	Heart disease
	Smallpox	Stroke
Cancer transition (and risk factor)	Stomach (*Helicobacter pylori*)	Lung, pancreas (smoking)
	Liver (hepatitis B and C viruses)	Breast (hormonal and physical activity)
	Cervical cancer (human papillomavirus)	Colorectum (Western lifestyle)

In all countries studied here, mortality from infectious-related cancers has declined steadily throughout the period, except for Japan (Figure 1). At the beginning of the study period, the age-standardized death rates from cancers associated with infectious causes ranged from 50.4 deaths per 100 000 population (95% CI, 49.9-50.8 deaths per 100 000 population) in the US to 159.0 deaths per 100 000 population (95% CI, 157.6-160.4 deaths per 100 000 population) in Japan, a ratio of 1 to 3.2. Note, however, that the series starts in 1959 in the US, but in 1950 for Japan. In Japan, the only country where mortality from these cancers increased, the peak was reached at 179.0 deaths per 100 000 population (95% CI, 177.6-180.5 deaths per 100 000 population) in 1956. At the end of the period (2016-2018, depending on the country), the rates for infectious-related cancers ranged from approximately 20 deaths per 100 000 population (21.2 deaths per 100 000 population in Norway [95% CI, 19.8-22.5 deaths per 100 000 population], 21.2 deaths per 100 000 population in the US [95% CI, 21.0-21.3 deaths per 100 000 population], 23.3 deaths per 100 000 population in England and Wales [95% CI, 22.9-23.7 deaths per 100 000 population], 24.2 deaths per 100 000 population in Sweden [95% CI, 23.2-25.2 deaths per 100 000 population]) to 62.1 deaths per 100 000 population in Japan (95% CI, 61.8-62.5 deaths per 100 000 population).

Stomach cancer contributed disproportionately to all infectious-related cancers in the 1950s (ranging from 70% in England and Wales to 80% in Norway, with the notable exception of the US, where it contributed <50%) and was associated with the overall decline of mortality from this type of cancer (Figure 2; eTable 1, eTable 2, eTable 3, eTable 4, eTable 5, and eTable 6 in the Supplement). Stomach cancer’s share was reduced to 20% to 30% (46% in Japan, where the rate has been much higher than elsewhere) by the end of the 2010s. The death rate from cervical cancer also exhibited a decline in all study countries throughout the period. Mortality from liver cancer did not exhibit any systematic trend across the 6 countries, with international variations in both levels and direction.

At the beginning of the study period, all countries exhibited an increasing trend in the noninfectious-related cancers. Every country but Sweden experienced a peak around 1990, with Sweden’s peak occurring in 1974. In all but the US and England and Wales, mortality from noninfectious-related cancers were at their lowest at the beginning of the study period. Initial rates varied more than for infectious-related cancers, ranging from 36.1 deaths per 100 000 population (95% CI, 35.4-36.8 deaths per 100 000 population) in Japan to 168.0 deaths per 100 000 population (95% CI, 166.5-169.6 deaths per 100 000 population) in England and Wales, a ratio of 1 to 4.7. The peak also varied from one country to another, with a minimum of 129.0 deaths per 100 000 population (95% CI, 128.3-129.7 deaths per 100 000 population) in Japan in 1996 up to 215.1 deaths per 100 000 population (95% CI, 213.7-216.6 deaths per 100 000 population) in England and Wales in 1985. Although the rates of noninfectious-related cancers show little signs of decline in Japan, rates for other countries have begun to decline.

In England and Wales and the US, trends in noninfectious-related cancers are largely determined by those in lung cancer, which plays a smaller role in the other countries (Figure 2). In most countries in our study, colorectal cancer is the second largest killer among the noninfectious-related cancers. Rankings of the other cancers in this category varies from country to country (eTable 1, eTable 2, eTable 3, eTable 4, eTable 5, and eTable 6 in the Supplement).

Japan and Norway are the only 2 countries where trends in mortality from the 2 groups of cancers intersect. Norway’s intersection in 1955 occurred earlier than Japan’s, which was in 1988.

## Discussion

In this study, we used temporal cancer-specific mortality data to assess whether the US, England and Wales, France, Norway, Sweden, and Japan have experienced comparable trends in cancer by type, albeit on a different schedule. Overall, there is compelling evidence supporting the cancer transition theory.

Of the more than 100 cancers responsible for the total cancer burden, the 9 cancers we examined account for more than two-thirds of all cancer deaths worldwide in 2018.^1^ In investigating the extent of the transition from infectious-related to noninfectious-related cancers, we have identified specific time points of convergence in Japan and Norway, with both countries exhibiting a crossover in trends from the 2 types of cancers during the study period. Visually, one way Japan’s trends are striking is that they intersect later in the period and most closely resemble an X pattern (Figure 1A).

Despite their considerable geographic, political, and cultural diversity, all 6 countries investigated here have experienced aspects of the transition sequence and appear to have been going through a systematic pattern of change in cancer mortality that can be summarized as follows: (1) initially, infectious-related cancer mortality rates are high and noninfectious-related cancer mortality are low; (2) then, infectious-related cancer mortality starts declining, while noninfectious-related cancer mortality increases; and (3) subsequently, infectious-related cancer mortality reaches a low level, while noninfectious-related cancer mortality starts declining after reaching its peak.

Of primary importance in our study is the grouping and analysis of infectious-related vs noninfectious-related cancers to identify signs of cancer transition. A caveat to such an analysis is the extent to which the overall trends mask some of the underlying diversity in specific cancers. Still, despite concealing heterogeneity across countries, grouping cancers by more or less infectious-oriented causes helps to reveal a concise picture of the cancer transition.

To conceptualize the cancer transition that occurs as nations develop, we apply elements of Omran’s theory of the epidemiological transition.^7^ Omran proposed 3 stages in the epidemiological transition, the last being a move to the age of degenerative and lifestyle-related diseases where illnesses such as stroke and cancer rise in prominence, while infectious diseases like malaria, tuberculosis, and smallpox become less common. We consider Omran’s understanding of cancer at the time as a noncommunicable or noninfectious-related disease, the classic view of cancer’s causes. In the last stage explicated by Omran, infectious diseases are increasingly brought under control, but not entirely. Historically, acute and infectious diseases tended to strike at younger ages, and when these diseases were brought under better control, people lived to increasingly older ages when noncommunicable, chronic diseases tended to strike.

Some researchers have attempted to extend Omran’s 3-stage theory by proposing a fourth stage or even fifth stage of the epidemiological transition.^8,37,38^ For instance, Olshansky and Ault^38^ postulated that there is a fourth stage of the transition that they term the “age of delayed degenerative diseases” in which major causes of death are still with us, but the risk of dying from those illnesses is shifted to older ages. Like many others during their time and continuing to this day,^8,9,10^ Olshansky and Ault adopt the classic view of cancer as a noncommunicable disease.

Emerging mainly from the anthropological literature is the idea that we are in a third transition, not to be confused with the stages of Omran’s epidemiological transition.^39,40^ According to this literature, the first transition is one that predates the first stage described by Omran, and occurred in the Neolithic period about 10 000 years ago. Authors writing on this topic also state that at about this time there was a shift marked by the emergence of many infectious diseases. The whole of Omran’s epidemiological transition is considered to be the second transition, and in the third transition there are emerging infectious diseases (eg, HIV, ebola virus, and COVID-19) and a reemergence of infectious diseases (eg, tuberculosis), many of which are antibiotic resistant.

Even with emerging diseases, deaths due to cancer, in the US at least, are far greater than those caused by acute infections. At the time of the writing of this study, COVID-19, which was first identified in December 2019, had killed approximately 400 000 Americans,^41^ but scientists have recently developed effective vaccines against the disease. Influenza and pneumonia, which kill people year after year and are also infectious, caused approximately 55 000 deaths in the US in 2017.^41^ In contrast, approximately 600 000 people died of cancer over the same year,^42^ and deaths due to acute infections have only accounted for a small portion (5.4%) of total deaths during the period between 1980 and 2014.^43^ These figures, along with the fact that many so-called new infectious diseases are actually hundreds if not thousands of years old,^44^ argue against a new stage of the epidemiological transition based on emerging and reemerging infectious diseases.

Although some proposed extensions to Omran’s epidemiological transition^7^ have made useful contributions to the literature, they do not grapple with the cancer transition theory as we have laid out in this study. Scholars have generally overlooked the fact that a number of widespread cancers are mainly caused by infectious agents. To wit, *H pylori* is a cause of stomach cancer, the human papillomavirus is a necessary, albeit insufficient, cause of cervical cancer, and hepatitis B and C viruses are causes of liver cancer.^13^ One major difference between Omran’s infectious diseases and the diseases associated with infection identified in our study is that the former are typically acute in nature whereas the latter are chronic and tend to develop over extended periods of time. As a consequence, the change in the mix of cancer mortality, with a shift from infectious causes to lifestyle-associated factors such as smoking, has not been temporally nested within the epidemiological transition. The cancer transition does not precisely mirror the epidemiological transition, but it is similar in the sense that the leading causes of mortality have been shifting from those associated with infections to those associated with other factors, prominent among which are individual behaviors such as smoking, drinking, diet, and physical activity.

With regard to *H pylori*, more than half of the world’s population in 2015 was infected with the bacterium,^45^ but only a small fraction of those infected will go on to develop stomach cancer.^13^ The prevalence of *H pylori* is declining in most countries of the world^46,47^ not through any specific medical measures, but as a result of general improvements in sanitation, housing conditions, access to clean water, food availability and freshness, and reduced salt intake.^14,48,49^

The human papillomavirus is transmitted sexually, with the majority of sexually active individuals of both sexes acquiring it at some point during their lifetime.^50^ Prior to the vaccine becoming available in 2006,^51,52^ screening and treatment were the only means to reduce cervical cancer incidence and mortality.^53^ Research is continuing into creating more affordable vaccines, which will especially help less developed countries where current levels of vaccination are a fraction of what they are in more developed countries.^52,54^ Reductions of cervical cancer will only be evident decades after vaccination, but a surrogate for cervical cancer—high-grade cervical intraepithelial neoplasia—has been substantially reduced in populations with high vaccine coverage.^55^

The last cancer associated with infectious causes that we investigated is liver cancer, and persistent hepatitis B and C viruses together account for approximately 90% of hepatocellular carcinoma, the most common form of liver cancer.^56^ A vaccine for hepatitis B has been available since the early 1980s, and was progressively administered to infants as part of national childhood immunization programs in 90 countries in the late 1980s and 1990s. Hepatitis B decreases have been observed in most countries.^57,58^ Currently there is not a vaccine for hepatitis C, but there is antiviral treatment, which cures approximately 90% of those infected with the virus.^59^ To summarize, there are reasons to expect that mortality from cancers associated with infectious causes will decline even further in the future in high- to low-income countries.

As for the combined noninfectious-related cancers, we found a plateauing or decline in 5 of the 6 study countries starting between 1985 and 2000. The pattern of risk factors over the last few decades in these countries for the individual cancers are mixed. A decline in total physical activity has been documented in Japan^60,61^ and the US.^62^ Although there does not appear to be country-specific data for the other nations we examine, one study found that in high-income Western countries, between 2001 and 2016, physical inactivity increased by 5%.^63^ Another important trend is that Japan and France seem to have largely resisted a degradation of their diets,^64,65^ but not the US^66^ and England and Wales.^67^ There is not enough literature to determine the extent to which food consumption has changed in Norway and Sweden.

The mean body mass index (BMI) in 2014 for Japanese men and women was in the normal range, but for the other countries, mean body mass indexes are in the overweight category for at least one sex, if not both.^68^ Alcohol consumption from 1990 to 2016 nearly leveled in the US and Japan, increased slightly in England and Wales, Norway, and Sweden, and declined markedly in France.^69^ In the countries we examine, smoking prevalence has declined, which has resulted in, or which is expected to result in, lower lung cancer rates with a 30- to 40-year lag.^70^ Sweden has had the greatest decrease in annualized rate of change of smoking prevalence between 1990 and 2015, whereas British men and French women have made the least amount of progress.^71^

Importantly, the ability to both detect and treat some cancers has improved dramatically, such as for cancers of the breast^72^ and colorectum.^73^ Prostate-specific antigen screening remains controversial,^74,75^ but treatment for prostate cancer has become more effective.^76^ When esophageal cancer is detected early, evolving therapies have improved the cure rate.^77^ For pancreatic cancer, mortality rates have decreased little despite advances in imaging and surgery.^78^

Considering these somewhat conflicting changes in the underlying factors associated with noninfectious causes of cancer, it is difficult to predict how fast mortality will decline in the future and how low a level it will ultimately reach. However, we found no reason to expect a reversal of their favorable trends in the study countries.

Our study’s findings support and extend the hypothesis of Gersten and Wilmoth^14^ that the cancer transition can be considered analogous to the epidemiological transition. Put another way, one can say that the cancer transition parallels the epidemiological transition in that there is an epidemiological transition also within the complex system of cancers linked to specific causes. This linkage between the epidemiological and cancer transition theories is presented schematically in the Table.

A main implication of our analysis, then, is that although Omran’s theory^7^ does not apply to part of the third stage of cancer transitions, in which noninfectious-related cancers have peaked and have, in some settings, begun to decline, cancer remains bound in epidemiological transition theory. Indeed, the theory continues to remain a useful framework for understanding fundamental mortality patterns for cancer, a leading cause of death worldwide.

### Limitations

This study had some limitations. Our analysis was constrained by the lack of comparable cause-of-death statistics for the years before the 1950s. Such data might have allowed us to capture the transition point in the 4 study countries (other than Japan and Norway) where it might have occurred earlier. In addition to issues of access to the necessary data, the fundamental changes in the *International Classification of Diseases* implemented between the fifth and sixth revisions (published in 1949) introduced important disruptions in historical cause-specific mortality series and could have made it difficult to follow trends in particular cancers.

## Conclusions

We examined cancer mortality trends in the US, select European nations, and Japan, and found substantial support for the cancer transition theory. This theory claims that as nations develop, rates of infectious-related cancers decline, whereas rates of noninfectious-related cancers increase. These trends parallel, and show the continued relevance of, the originally formulated epidemiological transition theory, which has had considerable influence on a number of academic fields.

## 

## Notes

Supplement. **eTable 1.** Age-Standardized Death Rates and 95% Confidence Intervals for Cancers of Infectious Roots, Cancers of Noninfectious Roots, and All Cancers in the Study and Proportion of Each Cancer Type (Infectious- vs Noninfectious-Related) Attributable to Specific Cancer Within Each Type, By Calendar Year, France **eTable 2.** Age-Standardized Death Rates and 95% Confidence Intervals for Cancers of Infectious Roots, Cancers of Noninfectious Roots, and All Cancers in the Study and Proportion of Each Cancer Type (Infectious- vs Noninfectious-Related) Attributable to Specific Cancer Within Each Type, By Calendar Year, England and Wales **eTable 3.** Age-Standardized Death Rates and 95% Confidence Intervals for Cancers of Infectious Roots, Cancers of Noninfectious Roots, and All Cancers in the Study And Proportion of Each Cancer Type (Infectious- vs Noninfectious-Related) Attributable to Specific Cancer Within Each Type, By Calendar Year, Japan **eTable 4.** Age-Standardized Death Rates and 95% Confidence Intervals for Cancers of Infectious Roots, Cancers of Noninfectious Roots, and All Cancers in the Study and Proportion of Each Cancer Type (Infectious- vs Noninfectious-Related) Attributable to Specific Cancer Within Each Type, By Calendar Year, Norway **eTable 5.** Age-Standardized Death Rates and 95% Confidence Intervals for Cancers of Infectious Roots, Cancers of Noninfectious Roots, and All Cancers in the Study and Proportion of Each Cancer Type (Infectious- vs Noninfectious-Related) Attributable to Specific Cancer Within Each Type, By Calendar Year, Sweden **eTable 6.** Age-Standardized Death Rates and 95% Confidence Intervals for Cancers of Infectious Roots, Cancers of Noninfectious Roots, and All Cancers in the Study and Proportion of Each Cancer Type (Infectious- vs Noninfectious-Related) Attributable to Specific Cancer Within Each Type, By Calendar Year, US
